# Cell-Type-Specific Expression of STAT Transcription Factors in Tissue Samples from Patients with Lymphocytic Thyroiditis

**DOI:** 10.1007/s12022-012-9204-0

**Published:** 2012-04-17

**Authors:** Julia Staab, Peter J. Barth, Thomas Meyer

**Affiliations:** 1 Klinik für Psychosomatische Medizin und Psychotherapie, Georg-August-Universität, Von-Siebold-Str. 5, 37075 Göttingen, Germany; 2 Gerhard-Domagk-Institut für Pathologie, Westfälische Wilhelms-Universität Münster, 48149 Münster, Germany

**Keywords:** Lymphocytic thyroiditis, Hashimoto's disease, STAT, Stromal fibrosis

## Abstract

Expression of cytokine-regulated signal transducer and activator of transcription (STAT) proteins was histochemically assessed in patients diagnosed as having Hashimoto's disease or focal lymphocytic thyroiditis (*n* = 10). All surgical specimens showed histological features of lymphocytic thyroiditis, including a diffuse infiltration with mononuclear cells and an incomplete loss of thyroid follicles, resulting in the destruction of glandular tissue architecture. Immunohistochemical analysis demonstrated differential expression patterns of the various members of the STAT transcription factors examined, indicating that each member of this conserved protein family has its distinct functions in the development of the disease. Using an antibody that specifically recognized the phosphorylated tyrosine residue in position 701, we detected activated STAT1 dimers in numerous germinal macrophages and infiltrating lymphocytes as well as in oncocytes. In contrast, STAT3 expression was restricted to epithelial cells and showed a clear colocalization with the antiapoptotic protein Bcl-2. Moreover, expression of phospho-STAT3 was associated with low levels of stromal fibrosis, suggesting that STAT3 serves as a protective factor in the remodeling of the inflamed thyroid gland. Phospho-STAT5 immunoreactivity was detected in numerous infiltrating cells of hematopoietic origin and, additionally, in hyperplastic follicular epithelia. This tissue distribution demonstrated that activated STAT5 molecules participate in both lymphocytopoiesis and possibly also in the buildup of regenerating thyroid follicles. Taken together, the cell-type-specific expression patterns of STAT proteins in human lymphocytic thyroiditis reflect their distinct and partially antagonistic roles in orchestrating the balance between degenerating and regenerating processes within a changing cytokine environment.

## Introduction

Hashimoto's thyroiditis, one of the most prevalent chronic autoimmune diseases affecting humans, is caused by autoreactive T lymphocytes that infiltrate the thyroid gland and ultimately lead to its progressive destruction. Numerous clinical and experimental studies have suggested a breakdown of organ-specific immune tolerance and inappropriate immune reactions against cellular components of the thyroid gland as the etiological basis of the disease. Accordingly, the histological hallmark of Hashimoto's disease is a chronic loss of the follicular organ structure and its concomitant replacement by lymphoid infiltrates including the formation of germinal centers and fibrosis [1–4]. Typically, the disease is characterized by the presence of a diffuse thyroid goiter, although in later stages the gland may progressively shrink until completely atrophic. Patients with chronic autoimmune thyroiditis usually present with elevated serum titers of autoantibodies such as antiperoxidase and antithyroglobulin as well as increased serum concentrations of thyroid-stimulating hormone (TSH) which result from peripheral hypothyroidism [4]. Although the diagnostic criteria for lymphocytic thyroiditis describe a well-defined disease entity, its pathogenesis is only poorly understood. Particularly, the role of extracellular cytokines and their intracellular signal pathways involved in lymphocyte recruitment to sites of thyroidal inflammation are, in general, not well defined.

A variety of cytokines including pro- and anti-inflammatory interleukins (IL) and interferon-γ (IFN-γ) have been detected in surgical specimens from patients with Hashimoto's disease and in rodents with experimental autoimmune thyroiditis induced by immunization with thyroglobulin and Freund's adjuvant [5–9]. Cytokines execute their pleiotropic effects mainly through the activation of members of the class of signal transducer and activator of transcription (STAT) proteins. Upon binding of a distinct cytokine to its cognate membrane receptor, the receptor-associated Janus kinases (JAKs) become autophosphorylated and consecutively phosphorylate critical tyrosine residues on the cytoplasmic tails of the receptor, thereby generating docking sites for STAT proteins or other intracellular signaling molecules [10–12]. STAT proteins are recruited to the phosphotyrosine-containing motifs on the cytokine receptor tails via their Src homology 2 domain and, in turn, are phosphorylated on a signature tyrosine within their carboxy termini. The activated STAT molecules then enter the nucleus as tyrosine-phosphorylated dimers where they recognize palindromic DNA sequences in the promoter region of cytokine-driven genes to induce gene expression.

Diversity in cytokine signaling is provided by the specificity of receptor binding and the dimerization of tyrosine-phosphorylated STAT variants. In humans, seven different members of the evolutionary conserved protein family of STAT transcription factors have been identified. Despite considerable structural similarity, STAT1 and STAT3 produce opposing physiological effects: whereas STAT1 involved in interferon signaling induces antiproliferative and apoptotic responses, IL-6-mediated STAT3 activation promotes cellular proliferation [10]. STAT5a/b proteins have been identified as key signaling molecules in normal lymphocytopoiesis as well as mammary gland development and lactogenesis [13, 14]. However, their expression in lymphocytic thyroiditis has not been described so far.

This report describes the use of immunohistochemical methods to assess the cellular distribution of STAT proteins in thyroid tissue from patients with lymphocytic thyroiditis. The results presented here show that STAT proteins are differentially expressed in a cell-specific manner and that their activation patterns vary depending on the extent of local intrathyroidal inflammation.

## Materials and Methods

### Patient Cohort

Ten patients with lymphocytic thyroiditis, who were admitted to the University Hospital of Marburg, Germany, for surgical therapy of diffuse swelling of the thyroid gland, were enrolled in this study. Patients were considered eligible for this study when conventional staining of paraffin wax-embedded thyroid tissue obtained from (hemi)-thyroidectomy demonstrated histopathological features of lymphocytic autoimmune thyroiditis. Thorough clinical examination excluded other causes of thyroid lesions such as Graves' disease, granulomatous thyroiditis DeQuervain, or Riedel's thyroiditis. Serum samples were collected and tested for the presence of antiperoxidase and antithyroglobulin autoantibodies using enzyme-linked immunoassays. All participants in the study gave their signed informed consent to a histological examination of thyroidal specimens. The study protocol was approved by the local ethics committee.

### Immunohistochemical Staining

After surgical resection, thyroid tissue samples were routinely fixed in 10% neutral buffered formalin and embedded in paraffin. Serial sections of 10 μm were cut from paraffin-embedded tissue blocks and deparaffinized through a graded series of alcohol. Subsequently, the rehydrated sections were heat-treated for 15 min followed by a three-step immunohistochemical staining procedure. For immunohistochemical detection of phospho-STAT1, we used a rabbit polyclonal antibody, diluted 1:100 in 4% bovine serum albumin/phosphate-buffered saline (BSA/PBS), which specifically reacted with tyrosine-phosphorylated STAT1 (Cell Signaling). STAT1 antibody C-24 was obtained from immunized rabbits (Santa Cruz) and diluted 1:500 in 4% BSA/PBS. STAT3 and its tyrosine-phosphorylated variant were detected using rabbit anti-STAT3 antibody H-190 and goat anti-phospho-STAT3 antibody pSTAT3-Y705, respectively (both from Santa Cruz). STAT5a/b immunoreactivity was probed with rabbit polyclonal antibody N-20 (Santa Cruz). Labeling of phospho-STAT5 was performed using the rabbit antibody pSTAT5a/b-Y694 (Santa Cruz). Mouse monoclonal antibodies against Bcl-2 (clone C8/144B, M7103) and CD8 (clone 124, M0887) were both obtained from Dako. Detection of bound immunoglobulins was achieved with the ABC method employing biotinylated secondary antibodies and avidin–horseradish peroxidase complexes. Diaminobenzidine was used as a substrate for visualization of the enzyme reaction yielding a brown reaction product. Finally, the sections were counterstained with Mayer's hematoxylin.

### Pathological Examination

The staining intensity for each target antigen was evaluated using a four-point semiquantitative scoring system ranging from 0 (indicating virtually no immunostaining) to 3 (with the most prominent parameters). Additionally, the extent of parenchyma replacement by collections of mononuclear cells was graded from mild to marked. Mild infiltration was defined as less than 10% loss of parenchyma, moderate indicated a 10% to 50% replacement, and marked indicated a greater than 50% replacement by cell infiltrates [1]. Additionally, stromal fibrosis was assessed using a four-point scale ranging from no fibrosis (0) to mild (1), moderate (2), and severe fibrosis (3).

### Statistical Analyses

Epidemiological data are presented as mean values ± standard deviations. Correlations between the different parameters were calculated using Spearman's rank order correlation coefficients. Probability values of less than 5% (*p* < 0.05) were considered statistically significant. All statistical analyses were performed with SigmaStat Version 9.0 from Systat Software.

## Results

### Characterization of the Study Cohort

All patients (*n* = 10) included in this study were clinically characterized by the presence of goiter and showed histological features of Hashimoto's disease or focal lymphocytic thyroiditis. Eight out of ten study participants were females; the mean age of the study population was 39.9 ± 12.1 years. The majority of patients were euthyroid (nine out of ten patients). In three patients, suppressed thyrotropin concentrations below the normal cutoff were detected and, interestingly, we found that a pathological decrease in serum TSH levels was significantly associated with a lack of STAT3 expression in thyroid biopsies (*p* = 0.048). Two individuals had normal serum concentrations of free triiodothyronine (fT_3_) and thyroxine (fT_4_) but a reduced level of TSH. One patient had fT_3_ and fT_4_ levels within the normal range and a TSH level above the normal cutoff. Elevated serum levels of antithyroid microsomal antibodies were detected in four patients (up to 9,460 U/ml, normal range <35 U/ml), and three of them also had an elevated titer of antithyroglobulin autoantibodies (up to 720 U/ml, normal range <40). In two antibody-negative patients, histological examination demonstrated, in addition to typical lymphoplasmacytic infiltration, either a well-differentiated, minimally invasive follicular thyroid carcinoma (case 2) or a follicular adenoma surrounded by fibrotic material (case 3). The characteristics of the study population including the results from laboratory measurements are presented in Table 1.

**Table 1:** Clinical characterization of 10 patients with chronic lymphocytic thyroiditis including assessment of histopathological features

Number	Age	Sex	Serum levels	Infil	Fibr	CD8	Bcl2	S1	pS1	S3	pS3	S5	pS5
1	64	f	fT3: 6.1	2	3	2	2	3	2	0	0	1	1
			fT4: 14.0										
			TSH: 0.03										
			Tg-Ab: <40										
			TPO-Ab: <35										
2	29	f	fT3: 5.2	3	3	2	2	3	1	1	0	1	1
			fT4: 13.4										
			TSH: 3.63										
			Tg-Ab: n.d.										
			TPO-Ab: n.d.										
3	38	m	fT3: 6.0	3	2	2	3	3	2	2	1	2	3
			fT4: 10.0										
			TSH: 1.9										
			Tg-Ab: n.d.										
			TPO-Ab: n.d.										
4	41	f	fT3: 7.8	3	3	2	2	1	1	0	0	2	1
			fT4:20.0										
			TSH: 0.01										
			Tg-Ab: 720										
			TPO-Ab: 894										
5	34	f	fT3: 4.5	2	3	2	2	2	1	0	0	1	1
			fT4: 9.9										
			TSH: 1.4										
			Tg-Ab: <20										
			TPO-Ab: 1,150										
6	41	f	fT3: 4.6	3	2	3	2	3	1	0	3	1	0
			fT4: 5.8										
			TSH: 17.0										
			Tg-Ab: 83										
			TPO-Ab: 9,460										
7	35	f	fT3: 5.2	1	2	2	2	3	2	1	3	2	0
			fT4: 17.0										
			TSH: 3.0										
			Tg-Ab: 32										
			TPO-Ab: 55										
8	20	f	fT3: 4.9	2	3	2	1	3	1	1	1	2	1
			fT4: 13										
			TSH: 1.3										
			Tg-Ab: 117										
			TPO-Ab: 607										
9	47	m	fT3: 4.8	1	2	2	2	3	1	1	2	2	2
			fT4:10.0										
			TSH: 4.9										
			Tg-Ab: <40										
			TPO-Ab: <35										
10	50	f	fT3: 4.8	1	3	2	3	1	1	0	1	3	0
			fT4:15.0										
			TSH: 0.13										
			Tg-Ab: n.d.										
			TPO-Ab: n.d.										

Histopathological assessment of stromal fibrosis, infiltration, and antibody staining was determined on a four-point scale ranging from 0 to 3 (0 = no, 1 = mild, 2 = moderate, and 3 = severe). Normal ranges for the indicated laboratory values are as follows: fT3 3.1–6.5 pmol/l, fT4 7.5–21 pmol/l, Tg-Ab <40 U/ml, TPO-Ab <35 U/ml, and TSH 0.34–5.6 mU/l.

*Bcl2* Bcl-2 expression, *Fibr* degree of stromal fibrosis, *Infil* degree of infiltration, *fT3* serum concentration of free triiodothyronine, *fT4* serum concentration of free thyroxine, *n.d.* not determined, *pS1* phospho-STAT1, *pS3* phospho-STAT3, *pS5* phospho-STAT5, *S1* STAT1, *S3* STAT3, *S5* STAT5, *Tg-Ab* serum titers of antibodies against thyroglobulin, *TPO-Ab* serum titers of antibodies against thyroid peroxidase, *TSH* thyroid-stimulating hormone

### Histological Examination of Thyroid Samples

**Fig. 1:**
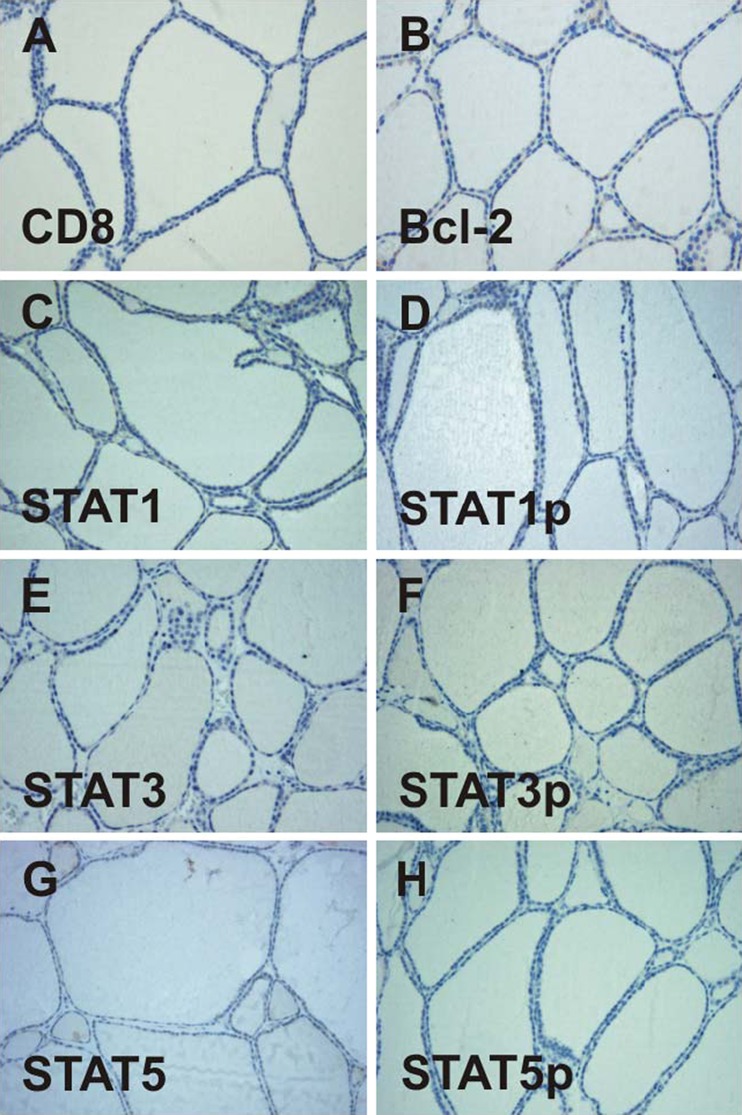
Immunohistochemical analysis fails to mark detectable amounts of CD8 (**a**), Bcl-2 (**b**), or the signal transducer and activator of transcription (STAT) proteins STAT1 (**c**), STAT3 (**e**), and STAT5 (**g**) including their corresponding tyrosine-phosphorylated protein modifications (**d**, **f**, **h**) in noninflamed thyroid tissue

In immunohistochemical specimens from normal thyroid tissue used as controls, colloid-containing follicles were evenly distributed in a fine interstitial stroma free of infiltrating CD8-positive cells (Fig. 1a). The prosurvival protein Bcl-2 was rarely detectable in normal glandular tissue (Fig. 1b). Furthermore, in controls without mononuclear infiltration, there was no immunoreactivity above the detection threshold for the following three STAT proteins tested: STAT1, STAT3, and STAT5 (Fig. 1c, e, g). Consequently, we did not detect tyrosine-phosphorylated forms thereof in normal tissue (Fig. 1d, f, h).

**Fig. 2:**
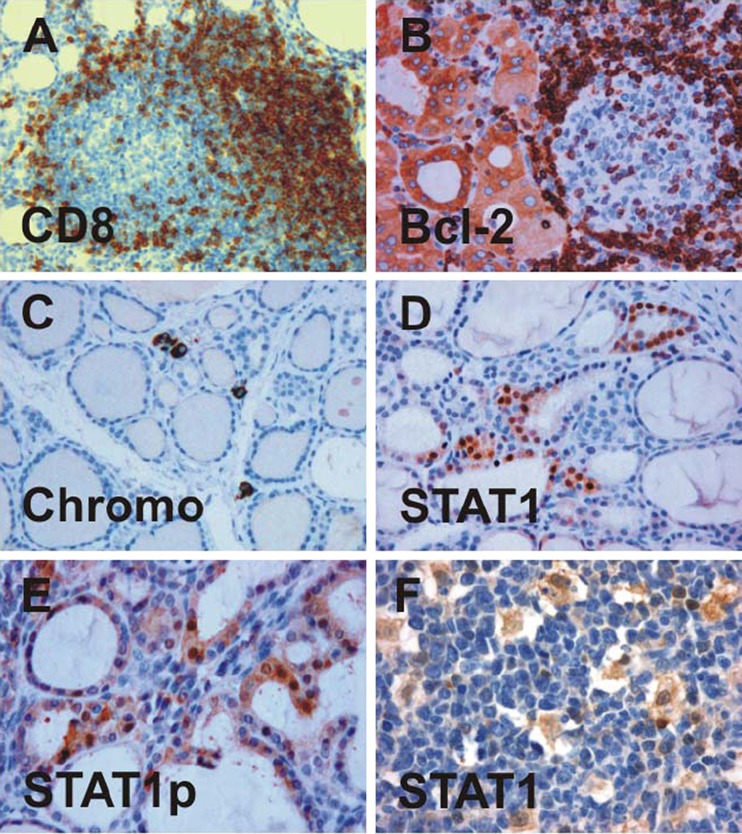
Expression of CD8 (**a**), Bcl-2 (**b**), chromogranin A (**c**), STAT1 (**d**, **f**), and tyrosine-phosphorylated STAT1 (**e**) in surgical samples from patients with lymphocytic thyroiditis as assessed by means of immunohistochemical staining using specific antibodies. The nuclear accumulation of phospho-STAT1 in epithelial cells lining thyroid follicles (**e**) and STAT1 localization in macrophages scattered throughout the center of a germinal lymph node (**f**) is shown

Tissue specimens from all study participants showed the presence of diffuse infiltrates of mononuclear cells localized between thyroid follicles; some of these cells stained positively for CD8 (Fig. 2a) or Bcl-2 (Fig. 2b), thus confirming the initial clinical diagnosis of Hashimoto's disease or lymphocytic thyroiditis [15, 16]. Morphological alterations in the follicular epithelium ranged from minimal irregularities in the shape of thyrocytes and degree of papillary folding, mainly observed in regions with mild infiltration, to a massive tissue remodeling characterized by pronounced atrophy of the follicular epithelium. The degree of follicular destruction varied according to the extent of lymphocytic infiltration, ranging from patchy to near complete effacement of the glandular architecture. In cases of severe interfollicular infiltration, thyrocytes in the vicinity of lymphoid infiltrates often displayed a swollen, oxyphilic, and granular cytoplasm. Generally, epithelial oxyphilia was associated with a significant decrease in thyroid parenchyma due to stromal fibrosis, which was accompanied by a nearly total depletion of colloid material in the remaining follicular lumen. The extent of parenchyma loss was less pronounced in tissue samples showing predominant hyperplastic changes in the follicular epithelium. Details on the histopathological features of biopsies from participants in the study are summarized in Table 1.

In cases of chronic inflammation, follicular cells exhibited a more or less cuboid or even slightly columnar morphology, while mononuclear cell infiltration and stromal fibrosis were relatively mild. Lymphoid follicles with activated germinal centers were present in all samples and were surrounded by damaged tissue showing follicular atrophy, colloid microfollicles, and occasionally, intrafollicular macrophages. The majority of immune-competent cells in the germinal center lacked expression of CD8 (less than 1% CD8-positive cells), whereas up to one-third of infiltrating cells scattered throughout the inflamed tissue stained positively for this marker (≤34% CD8-positive cells). In three patients, chromogranin A staining revealed rare calcitonin cells, which were arranged in small groups or single cells in the parafollicular interstitium (Fig. 2c).

### STAT1 Expression in Lymphocytic Thyroiditis

Using similar staining protocols and the same set of anti-STAT antibodies as described, we observed a cell-type-specific and disease-associated expression of STAT proteins in samples from patients clinically diagnosed as suffering from lymphocytic thyroiditis. Most prominent was a positive immunostaining for STAT1 transcription factor in the epithelial lining of structurally altered thyroid follicles, as was visualized in all surgical specimens tested (Fig. 2d). Atrophic follicles containing sparse colloid within a small and irregular lumen frequently showed a positive staining pattern with anti-STAT1 antibody, particularly when embedded in a dense lymphoplasmacytic infiltrate. At higher magnification, STAT1-expressing thyrocytes usually showed characteristic features of an oxyphilic change such as a voluminous, granular cytoplasm and enlarged, centrally, or eccentrically located nuclei with prominent macronucleoli. Occasionally, hyperplastic epithelium also showed a diffuse staining pattern with STAT1-reacting antibodies, indicating that STAT1 expression was not restricted to follicular cells within an oxyphilic-changed epithelium. Interestingly, STAT1 immunoreactivity in thyrocytes was higher in the nucleus as compared to the cytosolic compartment, indicating that activated STAT1 molecules were retained in the pleomorphic nuclei of oncocytic follicular cells. This nuclear accumulation of STAT1 in epithelial cells was confirmed with a polyclonal anti-STAT1 antibody, which specifically recognized tyrosine-phosphorylated STAT1 dimers (Fig. 2e).

**Fig. 3:**
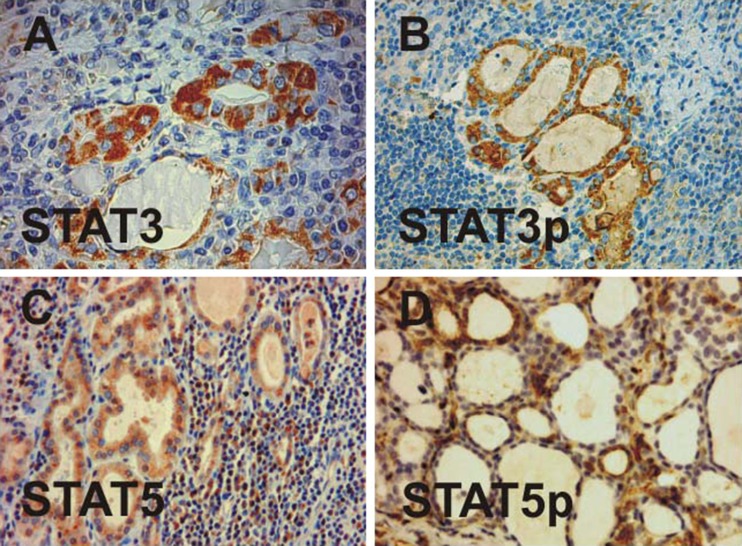
Detection of STAT3 (**a**), phospho-STAT3 (**b**), STAT5 (**c**), and phospho-STAT5 (**d**) in specimens from patients with lymphocytic thyroiditis as demonstrated by histopathological examination using appropriate primary antibodies

In addition to the epithelial lining of thyroid follicles, there was a strong expression of STAT1 in single cells scattered throughout areas of diffuse lymphoplasmacytic infiltration. These anti-STAT1-immunoreactive cells displayed a small rim of cytoplasm around their nuclei, suggesting that they were immune-competent cells of hematopoietic origin. Although readily detectable in the marginal zone surrounding activated lymphoid follicles, the density of these STAT1-expressing cells per microscopic field was not high as compared to cells expressing STAT5 (see Fig. 3c). Furthermore, numerous macrophages in the inner germinal center stained positively for STAT1, some of them exhibited engulfed phagocytic material within their prominent cytoplasm (Fig. 2f). Occasionally, phospho-STAT1-positive macrophages were also detected in the interior of thyroid follicles where they had lost contact to the epithelium (data not shown).

### Expression of STAT3 and Antiapoptotic Bcl-2

In five out of ten patients with chronic lymphocytic thyroiditis, we found intrathyroidal expression of STAT3, which was predominately located in regions with almost complete destruction of the follicular architecture. While STAT1 was expressed both in epithelial cells as well as infiltrating mononuclear cells, the cellular distribution of STAT3 was restricted to altered thyrocytes in residual follicles with oxyphilia (Fig. 3a). Also, in contrast to STAT1, we failed to observe nuclear accumulation of STAT3, even when using an antibody specifically recognizing tyrosine-phosphorylated STAT3 (Fig. 3b). Importantly, we found that activation of STAT3 was significantly associated with a lower degree of stromal fibrosis in the inflamed thyroid gland (*p* = 0.0015).

Since STAT3 and STAT5 are both known to be potent transcriptional activators of the *bcl2* gene, we next investigated the tissue distribution of this antiapoptotic protein. As expected, Bcl-2 immunoreactivity was observed in interfollicular mononuclear cells as well as in lymphoid follicles where the expression of Bcl-2 was predominantly restricted to B cells in the mantle zone and, to a lesser extent, to lymphocytes inside reactive germinal centers (Fig. 2b). In addition, we detected a significant Bcl-2 staining in thyroid follicles lined with oxyphilic epithelia, although the staining intensity in oncocytes was usually below that in the lymphocytes.

### Distribution of STAT5 and its Tyrosine-Phosphorylated Variant

**Fig. 4:**
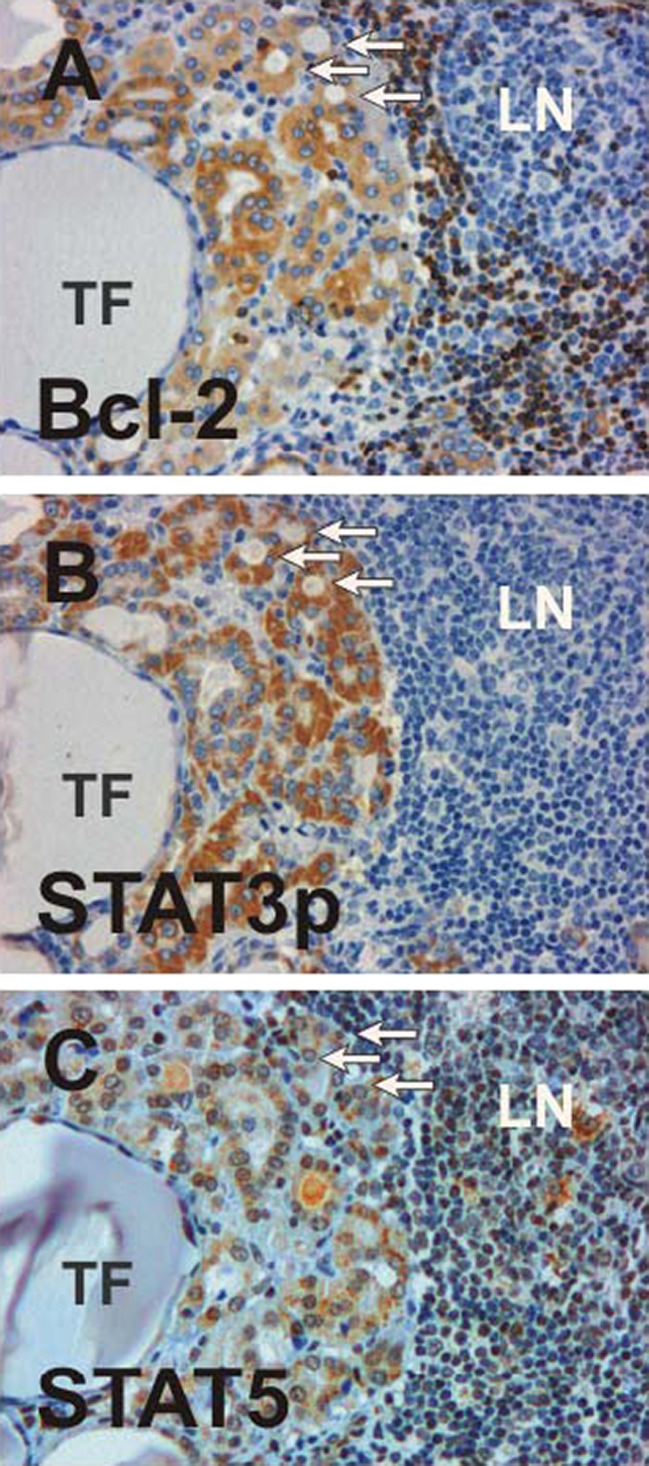
Colocalization of Bcl-2 (**a**), phospho-STAT3 (**b**), and STAT5 (b) in oncocytes as determined by staining serial sections with the corresponding antibodies. A cluster of three thyroid follicles (*TF*s) is marked with *arrows*. Note the absence of phospho-STAT3 expression in lymphocytes and the differential distribution of STAT5 and Bcl-2 in the constituting cells of the adjacent lymph node (*LN*)

STAT5a/b was preferentially expressed in specimens showing modest lymphocytic infiltration in areas with regenerating thyroidal colloids of reduced size (Fig. 3c). Nuclear accumulation was a hallmark of STAT5 distribution. Tyrosine-phosphorylated STAT5 molecules were also present in the cytosol, albeit in a lower concentration, as judged from the more intense nuclear staining (Fig. 3d). Similar to STAT1, anti-STAT5 antibodies reacted with both infiltrating mononuclear cells as well as follicular cells. However, we found neither statistically significant correlations between STAT1 and STAT5 expression nor between the phosphorylated STAT dimers thereof. Phospho-STAT5 expression was detected less frequently than STAT1, as only 70% of cases showed a clear STAT5 immunoreactivity. Epithelial STAT5 expression was most prominent in a follicular adenoma, which was diagnosed as an incidental finding (case 3). Serial sections revealed that Bcl-2, phospho-STAT3 and STAT5 were often coexpressed in the epithelial lining from the same thyroid follicles (Fig. 4). However, colocalization was not obligatory as Bcl-2-positive follicular structures frequently showed no staining with STAT5 antibodies.

## Discussion

In this study, we examined the expression of different STAT proteins in surgical samples from patients with lymphocytic thyroiditis, a disease characterized by diffuse infiltration of the thyroid gland with CD4^+^ and CD8^+^ T cells and plasmocytes. Our immunohistochemical data demonstrated that STAT1 transcription factor is abundantly expressed in the nuclei of oncocytes, also known as Hürthle cells, Askanazy cells, or oxyphilic cells, which are a hallmark of Hashimoto's disease, but not exclusively confined to this entity. There was evidence of a high level of tyrosine phosphorylation of STAT1 in these cells, as judged from staining with a phosphospecific STAT1 antibody which specifically reacted with the critical tyrosine residue in position 701 required for phosphodimer formation. Nuclear accumulation of STAT1 in the follicular epithelium was most prominent in areas displaying extensive disruption of the thyroid architecture due to the presence of a chronic inflammatory milieu. Tyrosine-phosphorylated STAT1 was also detected in macrophages located in intrathyroidal germinal centers, which displayed characteristic features of antigen-presenting cells such as phagocytosis of cell debris and an overall amoeboid morphology. The presence of phosphorylated STAT1 molecules in follicular macrophages is considered to be a marker for cell activation, pointing to the putative role of phospho-STAT1 as a disease-promoting factor in the orchestration of primary immune responses against thyroidal tissue.

Previous studies have shown that IFN-γ, a prototypic Th1 cytokine and potent activator of STAT1 tyrosine phosphorylation, stimulates macrophages and dendritic cells to induce MHC class II expression [17]. Interestingly, transgenic mice constitutively overexpressing IFN-γ specifically in thyroid follicle cells have been reported to develop primary hypothyroidism and growth retardation resulting from chronic, cytokine-mediated inflammation [8, 18]. Kimura et al. reported that in this transgenic mice line, thyroidal secretion of IFN-γ was associated with a transformation of normal thyrocytes into oncocytes [19]. When transgenic mice expressing IFN-γ in thyrocytes were crossed with RAG-2 knockout animals, which failed to generate mature T and B lymphocytes because of an inability to initiate the V(D)J recombination process, the architecture of the thyroid gland still remained disturbed despite the absence of infiltrating lymphocytes [18]. This observation suggests that local IFN-γ production in the thyroid gland per se causes the chronic hypothyroid phenotype independent of the cellular source of the cytokine [19–21].

When backcrossed in a NOD.H-2^h4^ background, the mice overexpressing IFN-γ in the thyrocytes failed to develop spontaneous thyroiditis, but instead showed a milder thyroidal inflammation and decreased frequency of activated CD44^+^ lymphocytes in cervical lymph nodes upon immunization with murine thyroglobulin and Freund's adjuvant [22]. Due to conflicting results, the significance of IFN-γ in the pathogenesis of experimental autoimmune thyroiditis still remains controversial [23, 24]. Additionally, one should be cautious in drawing conclusions from these animal studies as far as the phenotype of the human disease is concerned because in the transgenic mouse models, IFN-γ is produced ectopically by thyrocytes and not physiologically by infiltrating lymphocytes, which under usual circumstances, direct the dysregulated immune reactions against follicular autoantigens [18]. Our finding of high expression levels of phospho-STAT1 in the nuclei of oncocytes underlines the hypothesis that interferons most likely modulate the uniform morphological alterations in these cells via the induction of STAT1 tyrosine phosphorylation.

In contrast to the considerable expression of STAT1 in most of the samples examined, STAT3 was detected in only half of the study participants (five out of ten patients). Generally, expression of STAT3 and phospho-STAT3 was low and confined to restricted groups of adjacent thyroid follicles. Staining of consecutive tissue slides clearly demonstrated colocalization of either STAT3 or STAT5 with Bcl-2, confirming previous results that *bcl2* is a target gene of STAT3 and STAT5 transcription factors important for preventing apoptotic cell death [15, 25–27]. Whereas STAT3-expressing epithelia usually displayed Bcl-2 immunoreactivity, there were abundant follicles with Bcl-2 staining which lacked either STAT3 or phospho-STAT3 expression, indicating the presence of both STAT3-dependent and STAT3-independent pathways for *bcl2* gene induction.

Our finding that suppressed serum levels of TSH were significantly associated with a lack of STAT3 expression was not fully unexpected, since thyrotropin is known to induce transcriptional activation of STAT3 [28]. Previous studies have shown that upon stimulation of rat thyroid cells with TSH, STAT3, and not STAT1, is rapidly tyrosine phosphorylated, pointing to the role of STAT3 as a transcriptional activator for TSH-induced proliferative responses [29–31]. Our observation of a colocalization of STAT3 and Bcl-2 in a number of hyperplastic and oxyphilic thyroid follicles may indicate that STAT3 confers protection against cell death via transcriptional activation of antiapoptotic Bcl-2. Of course, from our small sample size comprising patients with chronic, long-lasting thyroiditis, we cannot exclude the possibility that thyroidal expression of STAT3 may be more prominent in earlier stages of the disease.

A central finding in our study cohort of patients with lymphocytic thyroiditis was that an increase in stromal fibrosis was associated with reduced levels of STAT3 activation. Furthermore, we found that STAT3 expression was restricted to epithelial cells and was completely absent in infiltrating T cells. These findings were unexpected, since STAT3 has been implicated in the development of autoimmune reactions. Interleukin-23 (IL-23) has been shown to stimulate naive CD4^+^ T cells via STAT3 activation to differentiate into IL-17-producing Th17 cells [32]. In turn, proinflammatory IL-17 induces the release of IL-6, which also upregulates prosurvival and proangiogenic genes through activation of STAT3 [32]. Our immunohistochemical results suggest that intrathyroidal proliferation of memory CD4^+^ T cells is not the key mechanism for STAT3 activation in lymphocytic thyroiditis and, moreover, that this transcription factor counteracts the autoimmune attack by reconstituting the inflamed organ via its direct effects on Bcl2-expressing thyrocytes. In this scenario, the glycoprotein 130–STAT3 pathway, known to be essential for Th17 development, may promote the growth and proliferation of regenerating thyroid follicles, thereby providing protection against an autoimmune destruction of the thyroid gland. Further experiments will have to elucidate the complex, and probably cell-type-specific antagonistic actions of the STAT3 transcription factor in the pathogenesis of this autoimmune disease.

The presence of STAT1 in all of our patients and its antagonistic counterpart STAT3 in half of the study population indicates the concurrent presence of both degenerative and regenerative processes during follicle remodeling. Cellular distribution of STAT5 was clearly distinguishable from the other two STAT proteins described above, since it was detected in numerous lymphocytes scattered throughout the intrathyroidal infiltrates and also in hyperplastic epithelium lining-affected thyroid follicles. STAT5 expression in infiltrating lymphocytes was expected because it is well known that activation of STAT5 in response to IL-7 receptor signaling facilitates cell survival in early B cell development and controls immunoglobulin gene rearrangements by suppressing premature Igk recombination in pro-B cells [14]. Surprisingly, however, activated STAT5 molecules were additionally found in numerous spheroidal thyroid follicles of reduced size that were tightly filled with colloid. This epithelial STAT5 expression in most, but not all, patients with lymphocytic thyroiditis resembles the histological features of the lobuloalveolar STAT5 distribution which have been reported for the normal development of the mammary gland during lactogenesis [13, 33]. Given the remarkable regeneration capacity of the human thyroid gland [2], we hypothesize that STAT5 is expressed mainly in regenerating thyroid follicles where it functions to restore the normal follicular structure of the organ. Further experiments will have to show whether indeed the course of murine experimental autoimmune thyroiditis is aggravated in transgenic mice lacking STAT5a/b expression in the thyroid gland.

Altogether, our immunohistochemical analysis in patients with lymphocytic thyroiditis demonstrated differential and cell-type-specific expression patterns for distinct members of the STAT protein family. Besides being expressed in a portion of infiltrating lymphocytes and germinal macrophages, tyrosine-phosphorylated STAT1 was associated with the transformation of normal follicular thyrocytes to typical oncocytes. In contrast, STAT3 expression was confined to epithelial cells and displayed clear colocalization with Bcl-2, suggesting that STAT3 executes protective functions in the tissue remodeling of the inflamed thyroid gland by upregulating antiapoptotic Bcl-2. STAT5 immunoreactivity was detected in both infiltrating cells of hematopoietic origin and hyperplastic follicular epithelium, demonstrating that STAT5 participates both in lymphocytopoiesis and possibly also in the buildup of newly formed follicle structures.
